# Is there scientific evidence of the mouthwashes effectiveness in reducing viral load in Covid-19? A systematic review

**DOI:** 10.4317/jced.57406

**Published:** 2021-02-01

**Authors:** Bianca L. Cavalcante-Leão, Cristiano-Miranda de Araujo, Isabela-Bittencourt Basso, Angela-Graciela-Deliga Schroder, Odilon Guariza-Filho, Glória-Cortz Ravazzi, Flavio-Magno Gonçalves, Bianca-Simone Zeigelboim, Rosane-Sampaio Santos, José Stechman-Neto

**Affiliations:** 1 Postgraduate Program in Communication Disorders, Tuiuti University of Paraná, Curitiba, Brazil; 2 Postgraduate Program in Dentistry, Pontifícia Universidade Católica do Paraná, Curitiba, Paraná, Brazil; 3 Department of Orthodontics, Pontifícia Universidade Católica do Paraná, Curitiba, Paraná, Brazil

## Abstract

**Background:**

The aim of this research is to verify whether there is evidence in the literature regarding the decrease in viral load present in saliva after using three types of mouthwashes.

**Material and Methods:**

Clinical and/or *in vitro* experimental studies that have used mouthwashes as a form of intervention to reduce the viral load in saliva were included. Combinations of words and appropriate truncations were adapted for each of the seven selected electronic bases including grey literature.

**Results:**

The selection of articles was carried out in two phases by two independent reviewers. After removing duplicate articles, 1245 references were maintained, and 2 articles were included in the Systematic Review. Both studies were performed in vitro and tested the virucidal action of the PVP-I solution for mouthwash at two different concentrations, 1% without dilution and 7% with 1:30 dilution, on the SARS-CoV and MERS-CoV viruses. Both showed a viral reduction of ≥ 99.99% with 15 s exposure.

**Conclusions:**

Based on the evidence currently available in the literature, PVP-I, at concentrations of 1 and 7%, appears to be the most effective mouthwash for reducing the viral load of COVID-19 present in human saliva. However, the guidelines for dental care refer to the use of hydrogen peroxide but there is insufficient scientific evidence to support this recommendation.

** Key words:**COVID-19, Coronavirus, Mouthwash, Chlorhexidine, Hydrogen Peroxide, PVP-I.

## Introduction

A recently published article mentions that dentists are the health professionals most at risk of being infected with COVID-19, with risks greater than that incurred by nurses and doctors; this is mainly due to the close relationship between professionals and patients (1). The transmission of the virus mainly occurs through inhalation/ingestion/direct contact of the mucosa with droplets of saliva, as it is known that this has an important role in the transmission of COVID-19 between people; these droplets are emitted during speech, coughing and/or sneezing (2,3).

Given the large number of infected people and the lack of effective treatments to combat COVID-19 thus far, measures have been taken to contain the spread of the disease based on past epidemic experiences with similar viruses, through hand hygiene, mask use, social distancing and oral hygiene by mouthwashes (4-6).

According to the latest information on COVID-19 (7), special attention has been given to dental risk, in which the asymptomatic patient has been mentioned many times, since the epithelial cells of the salivary glands, even the smallest ones distributed throughout the oral cavity, have high expression of the ACE2 receptor for COVID-19, surprisingly higher than the others found in the lungs. The contact between dental professionals and human fluids through direct or indirect contact shows a higher risk of infection since the oral mucosa is one of the first sources of contamination by coronavirus (8). Considering this form of transmission, some substances can be effectively used to decrease the viral load in saliva and oral mucosa, minimizing the risk of respiratory infections 1, 5.

Since the viral load contained in human saliva is very high, reaching up to 91.7% (9), mouthwashes with antiseptic rinses can not only reduce the amount of infection but also eliminate the virus in saliva and therefore facilitate the Fight against oral transmission (7,8,10). In some patients, SARS-Cov2 RNA was detected only in saliva and not in the respiratory tract. This form of infection is particularly relevant for health professionals who work in intensive care units and offices and professionals who perform bronchoscopies, endoscopies, or exams with close proximity to the patient, e.g., dentists (11-13).

Infection control measures are needed to prevent the virus from spreading and to help control the epidemic. There is still no systematic review that addresses the effects of mouthwashes against COVID-19, and it has been suggested that this virus can be transmitted by asymptomatic infection originating from contaminated saliva (9); therefore, this study aims to verify whether there is evidence in the literature regarding the effectiveness of three types of mouthwashes in decreasing the viral load present in the oral cavity; the three types of mouthwashes are used in dentistry:, chlorhexidine, hydrogen peroxide and povidone-iodine (PVP-I).

## Material and Methods

-Protocol and registration

This review was carried out by the Preferred Reporting Items for Systematic Reviews and Meta-Analysis Checklist (PRISMA) (14), and the protocol was registered on the website PROSPERO (International Prospective Register of Systematic Review - Centre for Reviews and Dissemination University of York) – CRD42020182213.

-Eligibility criteria

The acronym “PICOS” was used to consider the eligibility of studies for this review:

• *P* = population (individuals infected with coronavirus or the contaminated saliva of these individuals)

• I = intervention (Mouthwashes - Chlorhexidine, Hydrogen Peroxide and PVP-I)

• C = comparison (compared to a control group through a cross-sectional evaluation or compared to the same individual/saliva at first through a longitudinal evaluation)

• O = outcomes (viral infection or % of virus inactivation)

• S = study design (clinical trials or *in vitro* studies)

-Inclusion criteria

Clinical or *in vitro* experimental studies that used mouthwashes as a form of intervention as a hypothesis for decreasing the viral load in saliva were included. There was no restriction on gender, age, ethnicity of individuals, language of the study or time of publication.

-Exclusion criteria

The following exclusion criteria were applied: a) studies that have not evaluated coronavirus-infected individuals/saliva; b) studies where the evaluation was not carried out using saliva or where the evaluation was carried out on uncontaminated surfaces; c) studies that have not used mouthwashes as a form of intervention or that have used another treatment added to the use of the mouthwash; d) studies that have not evaluated the outcome of interest or that have incomplete data; and e) descriptive studies, such as reviews, letters, conference abstracts, expert opinions, and case reports.

-Information sources and search strategy

Combinations of words and appropriate truncations were adapted for each of the six electronic databases selected as information sources: PubMed/Medline, EMBASE, Latin American and Caribbean Literature in Health Sciences (LILACS), Web of Science, Scopus and Cochrane Library. Additionally, gray literature was used as a source of information through Google Scholar, Proquest and Open Gray (Table 1,  Table 1 cont.,  Table 1 cont.-1). Studies of electronic databases and gray literature were carried out on April 24, 2020, and all references were managed. All duplicate studies were removed using appropriate software (EndNote® X7 Thomson Reuters, Filadélfia, PA).

**Table 1:**
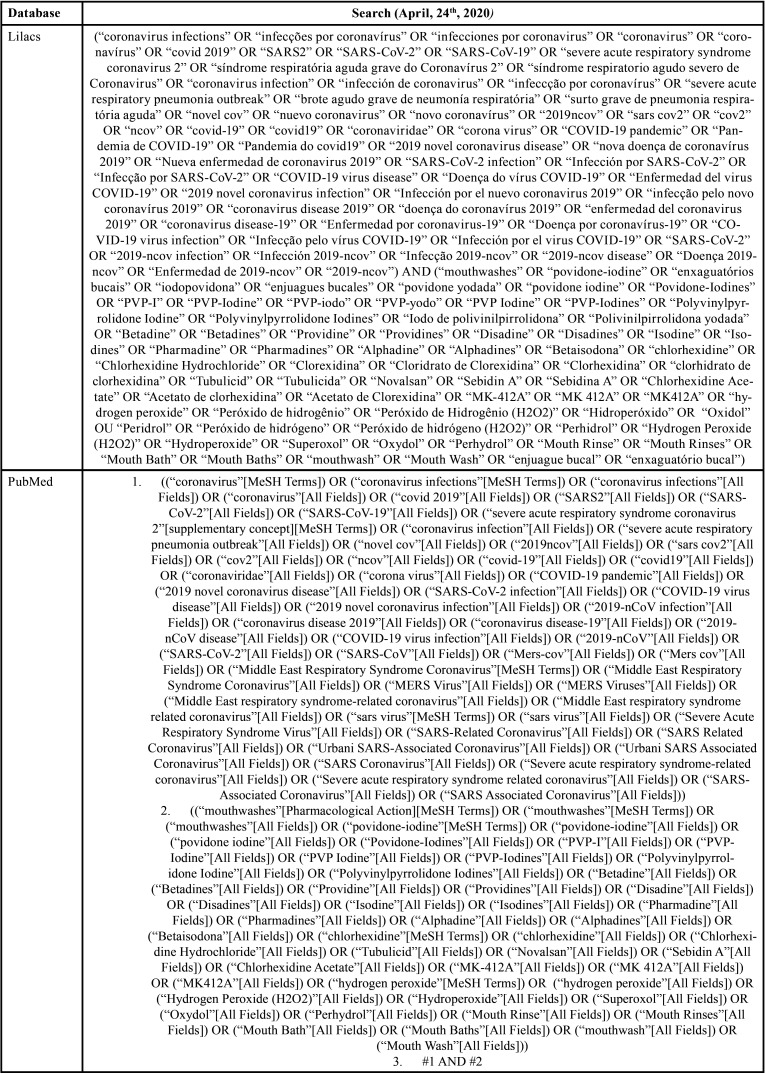
Database search strategy.

**Table 1 cont.**
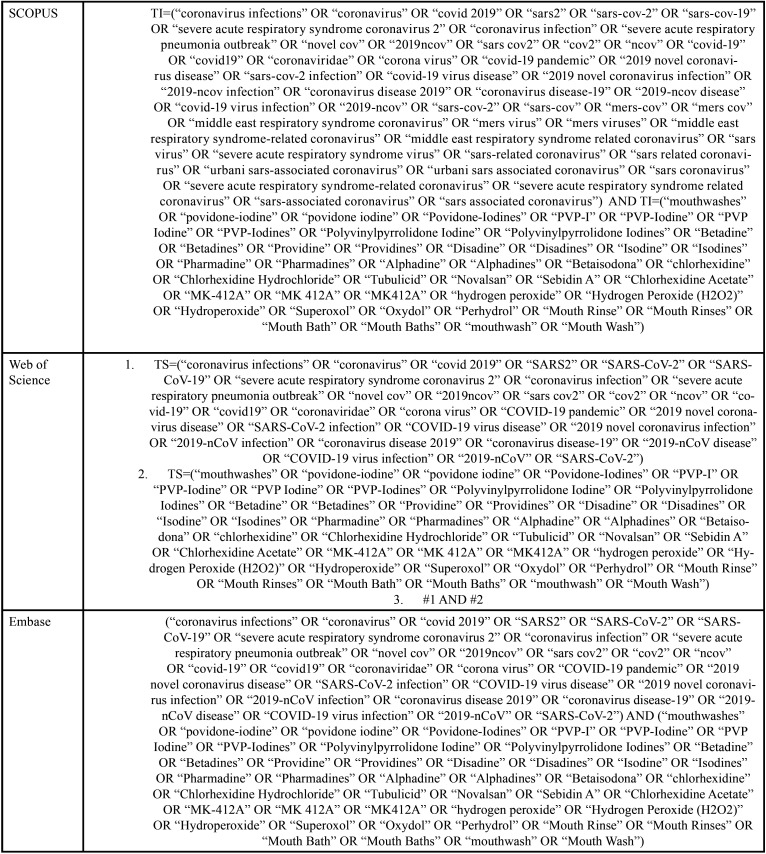
Database search strategy.

**Table 1 cont.-1:**
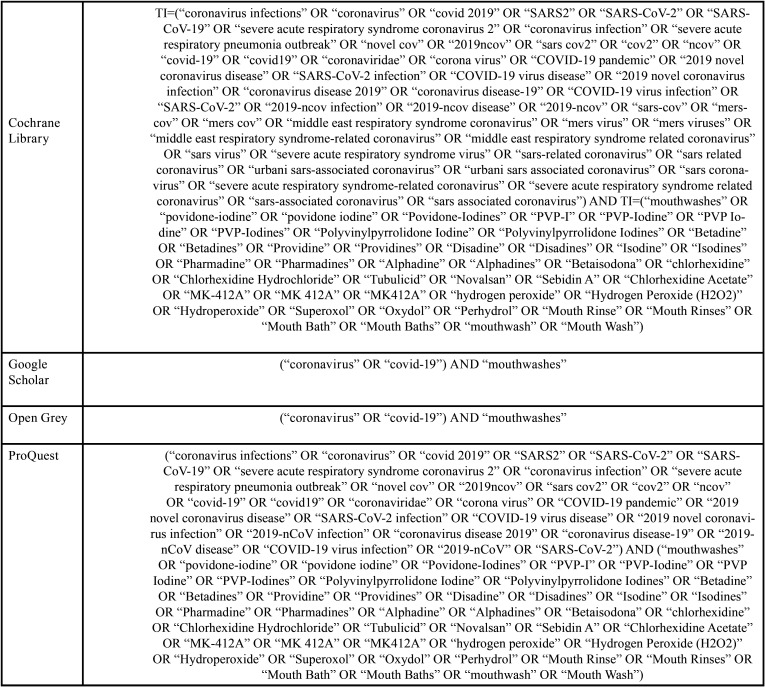
Database search strategy.

A manual search of references was carried out in all included studies and in the most current guidelines in the literature that have addressed forms of prevention in dentistry related to coronavirus. An expert on the subject was consulted via e-mail to verify any possible publication on the subject and indication of any relevant articles that could be included.

-Study selection

The selection of articles was carried out in two phases. In the first phase, two reviewers (B.L.C.L. and A.G.D.S.) independently reviewed the titles and abstracts of all references. All articles that did not meet the eligibility criteria previously established were excluded at this stage. In the second phase, the same reviewers independently read the full text of the articles selected in the first phase. Whenever there was some disagreement and a lack of consensus persisted even after discussion, a third reviewer (G.C.R) was involved in the final decision.

To facilitate reading independently in both phases, the website Rayyan (http://rayyan.qcri.org) was used, where the reviewers were shielded in all evaluations and a third member of the team (C.M.A.) acted as a moderator.

-Data collection process

Two reviewers independently (B.L.C.L. and A.G.D.S.) collected information from the included studies, and this information was discussed with two other team members (G.C.R and B.S.Z.). The collected data consisted of characteristics of the study (e.g., author, year of publication, country and study design), characteristics of the population (e.g., mouthwash used and protocol used), results and conclusion. When data were missing or incomplete in the article, attempts were made to contact the authors to obtain relevant unpublished information.

-Risk of bias in individual studies

For the evaluation of experimental studies in humans (clinical trials), the tool “Cochrane Collaboration tool for assessing the risk of bias” was used (15). This tool covers seven domains: 1) generation of the random sequence; 2) concealment of allocation; 3) blinding of participants and professionals; 4) blinding of outcome evaluators; 5) incomplete outcomes; 6) report of selective outcome; and 7) other sources of bias. The judgment regarding the possible risk of bias in each domain was made based on the information extracted from the study and was classified as “high risk”, “low risk”, or “not clarified” when there were not enough details reported in the study.

As there is no standard tool for risk assessment of bias for *in vitro* studies, the risk of bias analysis was performed based on an adaptation to another previous study (16). Two reviewers (I.B.B. and R.S.S.) independently assessed the included studies, classifying the quality of the report, performance bias, selection bias and detection bias. The risks of these different domains were labeled as having a low, unclear or high risk of bias.

-Summary measures

Any outcome measure was considered, provided that the outcome of interest was assessed.

## Results

-Study Selection

**Figure 1:**
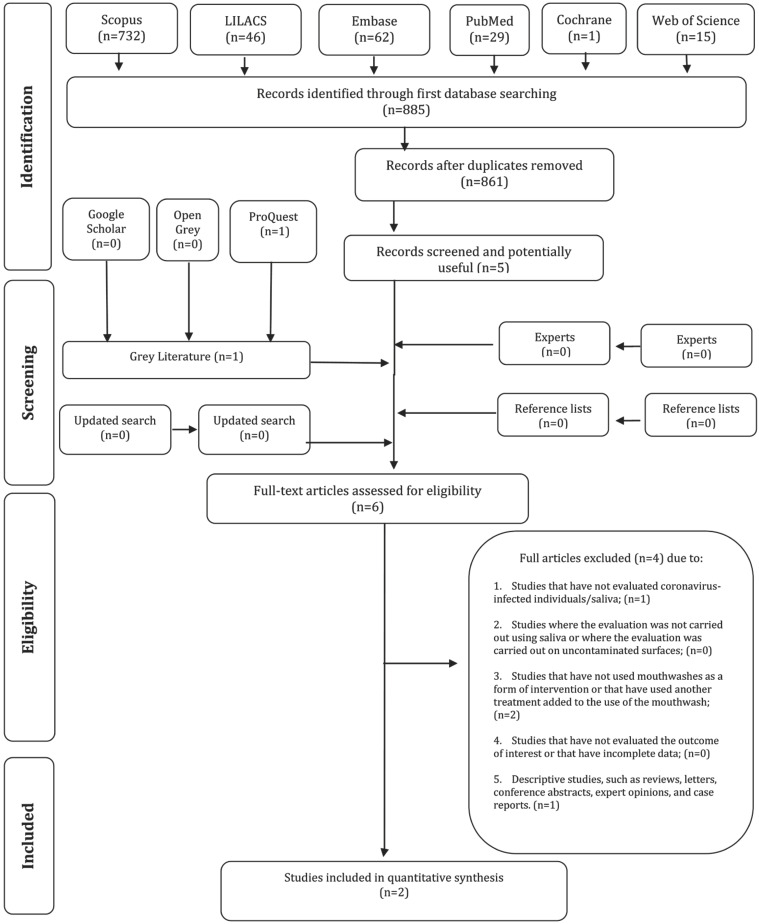
Diagram of Literature Search and Selection Criteria (1) Flow.

A total of 1222 articles were retrieved from the six electronic databases (PubMed/Medline, EMBASE, Latin American and Caribbean Health Sciences Literature -LILACS, Web of Science, Scopus, Cochrane Library). After removing duplicate articles, 1133 references were maintained. Subsequently, applying the eligibility criteria, 1124 studies were excluded, resulting in 9 articles. A search was performed in the gray literature, and one study was selected, thus totaling 10 articles for a complete reading. After the complete reading (second phase), eight articles were excluded (Table 2), resulting in two studies included for the qualitative synthesis of the results (Fig. 1). No additional articles were selected from the list of references or as indicated by the expert. Kappa coefficient of agreement index was > 0.8, indicating excellent agreement between the reviewers.

**Table 2:**
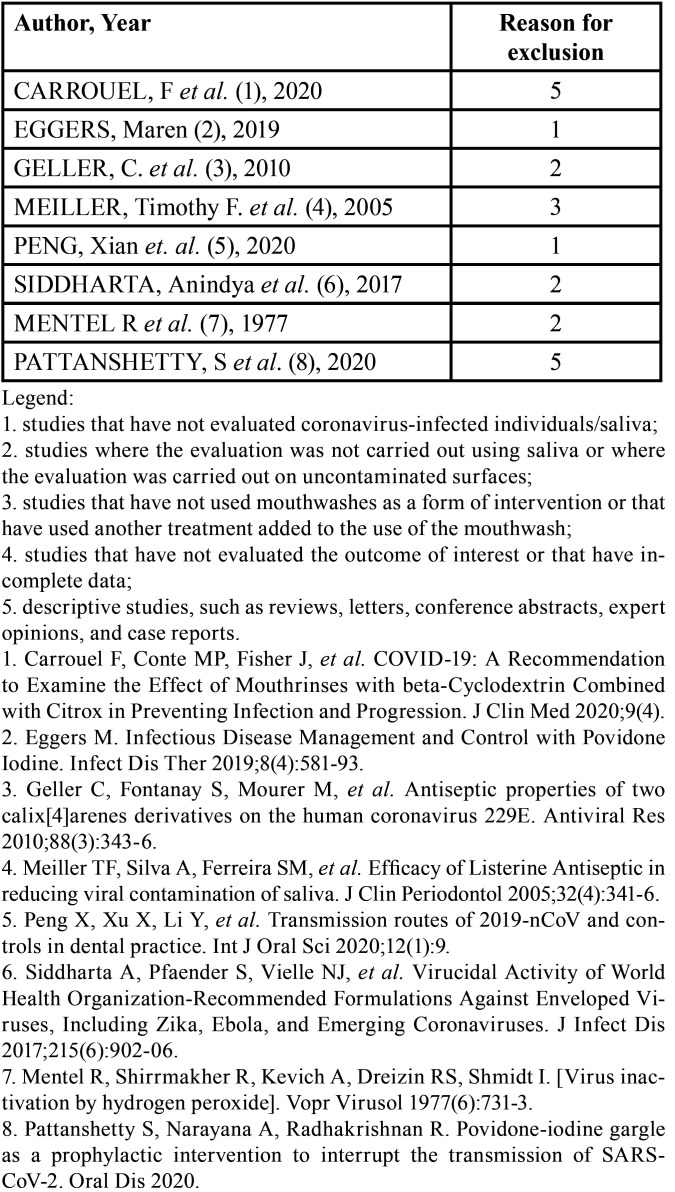
Excluded articles and reasons for exclusion (n=8).

-Study Characteristics

The two included studies were published in 2015 4 and 20185, and both were conducted in Germany. Both studies were performed *in vitro* and tested the virucidal action of the PVP-I solution for mouthwash at two different concentrations, 1% 4 and 7%5. The outcome evaluated in both studies was the viral titer of the MERS-Cov virus 4 and the MERS-Cov and SARS-Cov viruses 5, tested in environments with clean and dirty conditions.

The culture medium in these studies consisted of 0.3 g/L BSA bovine serum albumin for the clean condition and 3.0 g/L BSA + 3.0 ml/l erythrocytes as interference substance for the dirty condition.

No analytical study evaluated the virucidal action of chlorhexidine or hydrogen peroxide on coronavirus in saliva, and no included study evaluated the action of one of the 3 rinses on SARS-cov2.

-Risk of bias within studies

**Figure 2:**
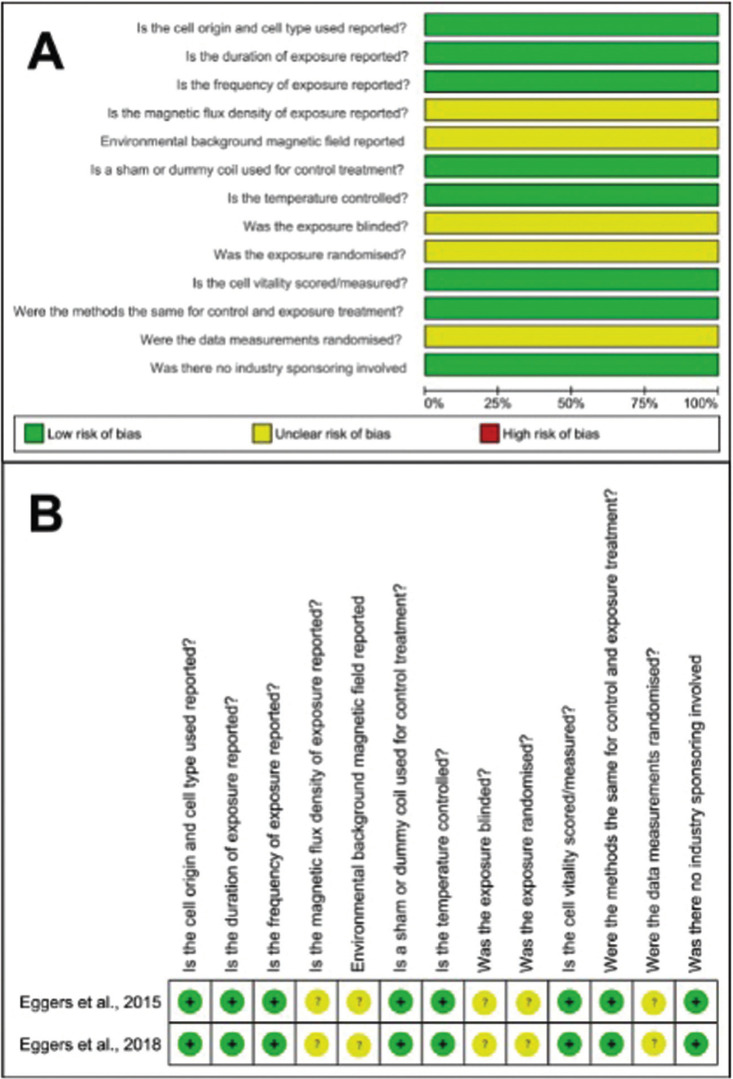
Risk of Bias.

Of the 13 items assessed for risk of bias, the 2 included studies were categorized as having a low risk of bias for 8 items. The domains that obtained the highest “unclear risk” rate (2 in each domain) were the quality of the report and the performance bias. None of the items in the domains evaluated in the two studies received a “high risk of bias” (Fig. 2, Table 3).

**Table 3:**
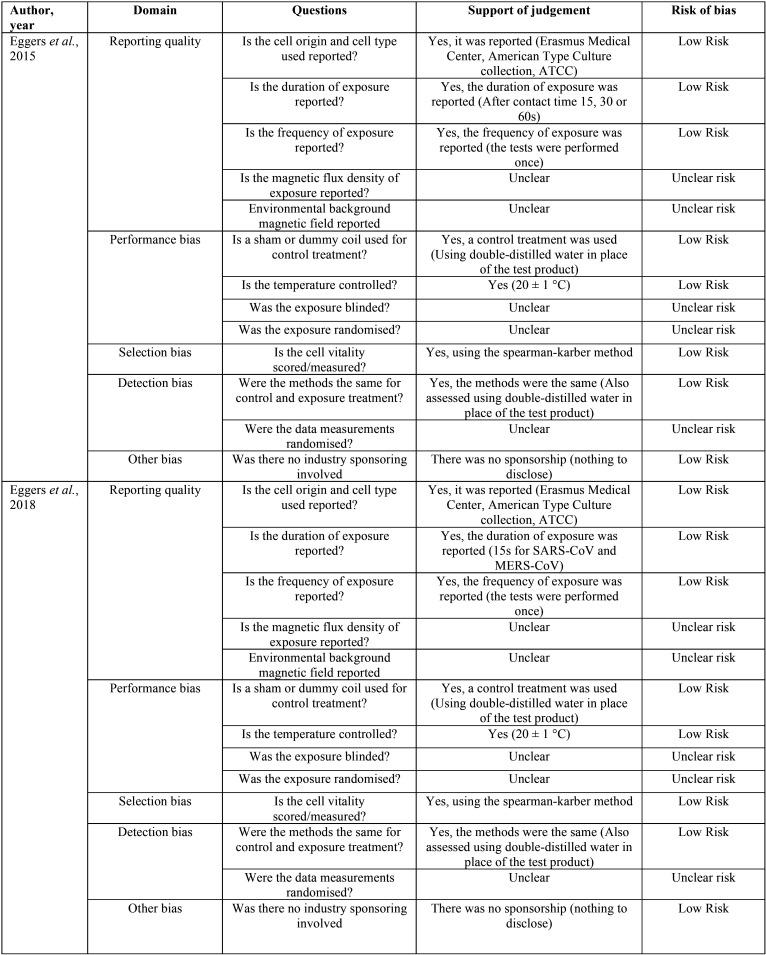
Excluded articles and reasons for exclusion (n=8).

-Results of individual studies

Eggers *et al.*, 2015 (4) and Eggers *et al.*, 2018 (5) performed *in vitro* tests to test the effectiveness of PVP-I at concentrations of 1% and 7% (with a 1:30 dilution resulting in a concentration of 0.23%) in the form of gargle/mouthwash on the MERS-Cov virus. In both studies, a viral reduction corresponding to ≥ 99.99% was achieved with 15 s exposure to the mouthwash agent. The titer of the MERS-Cov virus present in the sample was from 6.00 to 6.50 log10 TCID50/ml, and after 15 s, there was a reduction in 4.30 log10 TCID50/ml 4, with similar reductions for both SARS-Cov and MERS-Cov viruses 5.

## Discussion

It is known that COVID-19 is a disease easily transmitted by droplets of saliva from an infected individual, by direct contact on the oral, nasal or ocular mucosa, or indirectly on surfaces where small particles are spread in the air through coughing or sneezing., as well as via instruments that produce aerosols in rooms and enclosed spaces, such as in dental offices (1,5,8). It should also be noted that the virus can survive on hands, objects and surfaces that have been exposed to infected saliva for up to 9 days 1. Due to the characteristics of dental environments, the risk of cross-infection can be high between patients and dentists. For dental offices and hospitals in areas affected (potentially) by COVID-19, rigorous and effective infection control protocols are urgently needed (7). Based on the available literature, studies were sought to assess the efficiency of mouthwashes based on hydrogen peroxide, chlorhexidine and PVP-I as a way to decrease the viral load in saliva and thus to reduce the risk of contagion.

Preoperative rinses reduce the number of microorganisms in the oral cavity (8), and several types are commercially available; their action on the chemical control of dental plaque is already well known (17). Some studies suggest that the use of mouthwashes is the best method for preventing the transmission of viruses and bacteria (2,18). Among these chemical agents, not all have bactericidal or virucidal efficacy, and others have undesirable side effects (19).

To prevent the spread of the virus, the governments of some countries have recommended, among the methods of cleaning the environment, the use of hydrogen peroxide (20). This solution is already widely used as an environmental surgical disinfectant and as an oral disinfectant in the treatment of gingivitis, as reported in the literature (21,22). Another study performed in 2016 18 in hospitalized patients and under mechanical ventilation showed that the use of 3% hydrogen peroxide mouthwashes significantly reduced the incidence of pneumonia in these patients. Despite this finding, there are no studies to date that prove the efficiency of this substance in decreasing the viral load in saliva, despite its ability to serve as an excellent bactericide in patients with pneumonia associated with mechanical ventilation in hospitals (18).

As instructed by the Guideline for the diagnosis and treatment of new coronavirus pneumonia (5th edition) released by the National Health Commission of the People’s Republic of China, chlorhexidine, which is commonly used as a mouthwash in dental practice, is not effective in reducing the viral load of COVID-19 8. Since this virus is vulnerable to oxidation, pre-procedure mouthwashes containing oxidizers are recommended, such as those with 1% hydrogen peroxide or 0.2% PVP-I, to reduce the salivary viral load, including the potential transport of COVID-19 (8). This result is in line with the results obtained in the present systematic review, which point to the effectiveness of mouthwashes based on 1% PVP-I (without dilution) and 7% (diluted at 1:30), which significantly decrease the viral load in saliva. The *in vitro* study conducted by Eggers *et al.* (4) demonstrated that the use of 7% PVP-I in the dilution of 2 ml to 60 ml of water (1:30), thus making the concentration 0.23%, even for 15 s, is already sufficient to exterminate the bacteria and viruses present in the samples analyzed with the MERS-Cov and SARS-Cov viruses. Furthermore, oral care products based on PVP-I do not irritate the oral mucosa during prolonged use (5).

A standardization of protocols in the dental clinic is necessary to improve the quality of care for patients seeking treatment. The use of a mouthwash is essential to reduce COVID-19 person-to-person transmission. The mouthwash with a PVP-I solution with a concentration of 1% (without dilution) and one of 7% (diluted at 1:30) examined in this systematic review has a killing effect on bacteria and viruses.

The results of this systematic review, based on *in vitro* studies, indicate that the use of PVP-I at concentrations of 1% (without dilution) and 7% (diluted at 1:30) is more effective in reducing the viral load of the family coronavirus than other products, such as chlorhexidine and hydrogen peroxide. However, it is worth noting the limitations of this study design, requiring more research (mainly randomized clinical trials) using different concentrations, times of use, and effectiveness of such products on the COVID-19 virus.

-Conclusions and Practical Implications

Based on the evidence currently available in the literature, PVP-I at concentrations of 1% (without dilution) and 7% (diluted at 1:30) for 15 s seems to be the most effective mouthwash for reducing the viral load of COVID -19 present in human saliva. However, the guidelines for dental care refer to the use of hydrogen peroxide but there is insufficient scientific evidence to support this recommendation. The level of scientific evidence, related to the use of PVP-I mouthwash, is very fragile because it is two *in vitro* studies.
